# Effect of Stress Amplitude on the Damping of Recycled Aggregate Concrete

**DOI:** 10.3390/ma8085242

**Published:** 2015-08-14

**Authors:** Chaofeng Liang, Tiejun Liu, Jianzhuang Xiao, Dujian Zou, Qiuwei Yang

**Affiliations:** 1 Department of Civil Engineering, Shaoxing University, Shaoxing 312000, China; E-Mails: liangchaofeng@usx.edu.cn (C.L.); yangqiuwei79@126.com (Q.Y.); 2 Shenzhen Graduate School, Harbin Institute of Technology, Shenzhen 518055, China; E-Mail: zoudujian@163.com; 3 Department of Structural Engineering, Tongji University, Shanghai 200092, China; E-Mail: jzx@tongji.edu.cn

**Keywords:** stress amplitude-dependent damping, loss factor, recycled aggregate concrete, damping energy coefficient, damping energy exponent

## Abstract

Damping characterizes the energy dissipation capacity of materials and structures, and it is affected by several external factors such as vibrating frequency, stress history, temperature, and stress amplitude. This study investigates the relationship between the damping and the stress amplitude of environment-friendly recycled aggregate concrete (RAC). First, a function model of a member’s loss factor and stress amplitude was derived based on Lazan’s damping-stress function. Then, the influence of stress amplitude on the loss tangent of RAC was experimentally investigated. Finally, parameters used to determine the newly derived function were obtained by numerical fitting. It is shown that the member’s loss factor is affected not only by the stress amplitude but also by factors such as the cross section shapes, boundary conditions, load types, and loading positions. The loss tangent of RAC increases with the stress amplitude, even at low stress amplitude. The damping energy exponent of RAC is not identically equal to 2.0, indicating that the damping is nonlinear. It is also found that the energy dissipation capacity of RAC is superior to that of natural aggregate concrete (NAC), and the energy dissipation capacity can be further improved by adding modified admixtures.

## 1. Introduction

Damping reflects energy dissipation, and it is one of the intrinsic dynamic characteristics of a material. It strongly affects a structure’s dynamic response and damage evolution. Thus far, a uniform understanding of damping that explains its complex mechanism in detail has not been obtained. Concrete is a brittle material consisting of a three-phase medium: coarse aggregate, cement matrix, and interfacial transition zone (ITZ). Every component has a significant effect on the damping of concrete. Many studies have focused on the influence of admixtures on the damping of concrete; however, few studies have investigated the influence of coarse aggregate, especially recycled coarse aggregate (RCA). Chung *et al.* [1,2,3,4] studied the damping capacity and storage modulus of cement paste and mortar with silica fume, latex, methycellulose, and graphite as admixtures. Neithalath *et al.* [5] investigated the influence of morphologically altered cellulose fibers on the acoustic and damping characteristics of cement mortar. It should be noted that most studies focused on enhanced damping by admixtures at the material level. In contrast, Ou *et al.* [6,7] conducted experimental studies at the structural level. The dynamic response of reinforced concrete (RC) structures with vibration-reducing admixtures was studied by shaking table and uniaxial hysteretic experiments. Swamy and Rigby [8] and Jordan [9] found that the influence of the addition of aggregate inclusions on the damping capacity of the mortar matrix depended on the volume content of the aggregate, the degree of hydration, and the state of curing, and that the addition of coarse aggregate reduced the damping of mortar. Ke *et al.* [10] experimentally investigated the damping ratio of concrete cantilever and found that the damping ratio increased remarkably after the surface of natural coarse aggregates was covered with viscoelastic materials (e.g., asphalt), and concluded that the weaker interface between the treated surface of natural coarse aggregate and cement mortar increased the interfacial viscous sliding during vibration.

External conditions such as vibration frequency, stress history, temperature, and stress amplitude influence damping. Among these, stress amplitude is one of the important factors influencing damping, especially for concrete materials. Lazan [11] found that many building materials were not completely elastic even when their stress was low, and their stress-strain curves formed hysteresis loops. Through numerous experimental investigations, he provided a damping-stress function (Equation (A1)) that described the power relationship between the damping energy and the stress. Moreover, the damping energy coefficient and the exponent of the damping-stress function have been determined for some materials such as mild steel but not for concrete. Kume *et al.* [12] evaluated the loss factor of a cantilever steel beam in terms of Lazan’s damping-stress function. In their study, the stress distribution function was determined by the quasi-static deformation in the cantilever beam as given by the solution for undamped forced vibration. However, the stress distribution function can only be derived theoretically for simple structural members. Therefore, Gounaris *et al.* [13,14] combined the finite element method and the numerical iterative technique with Lazan’s damping-stress function to calculate the nonlinear material damping of complex members. Furthermore, the parameters of the damping-stress function for mild steel were determined by a newly developed method called the iterative complex eigensolution method.

Jordan [9] investigated the effect of stress, frequency, mix, and age on the damping of concrete and found that wet concrete showed a steady increase in damping with an increase in the maximum stress. Hwang *et al.* [15] experimentally determined the effect of the displacement history sequence and magnitude on the cyclic response of reinforced flexural members and found that the energy dissipation capacity was a function of the applied stress intensity and the magnitude of maximum displacements applied to the members. Darwin *et al.* [16] studied the energy dissipation index of RC beams with different geometries under cyclic load and found that it was primarily controlled by the maximum stress, concrete strength, and transverse steel capacity. Wang *et al.* [17] presented a new method to calculate the material damping of RC components in the elastic stage under axial cyclic load; furthermore, they established a relation formula for the energy dissipation of RC beams with maximum stress amplitude, concrete strength, and reinforcement ratio through nonlinear regression. Jeary *et al.* [18,19] employed a time series analysis method to obtain the relationship between structural damping and vibration amplitude at high amplitude level, and took the fracture at both microscopic and macroscopic scale into consideration. Li *et al.* [20,21] emphasized that the effect of amplitude-dependent damping on the dynamic responses of a super tall building was significant and that the knowledge of the actual damping characteristics was very important for the accurate prediction of wind-induced vibrations in such buildings.

The recycled aggregate concrete incorporates recycled aggregates produced from construction and demolition wastes, and it is an environment-friendly building material. Therefore, the use of recycled aggregates as structural grade concrete has generated extensive interest in civil engineering construction. However, the recycled aggregate always has a lot of mortar on the surface and internal micro-cracks [22,23]. The shape and amount of micro-cracks existing in recycled aggregate deteriorate the mechanical properties of RAC, such as compressive and tensile strength [24,25,26,27]. Due to the intrinsic porosity of RA, the durability of RAC is worse than that of NAC [28,29]. The creep and drying shrinkage of RAC increase significantly due to the lower elastic modulus of RA [30,31]. The fatigue life of RAC is lower than that of NAC for the same stress level under cyclic bend loading and the dynamic compressive strength decreases with an increasing RA replacement ratio at high strain rates [32,33]. The nature and initial moisture condition of RA determine the microstructural characteristics of the aggregate–cement paste interface and the mechanical properties of the ITZ [34,35]. Currently, a large number of studies have been carried out to evaluate the mechanical properties, durability, and volume stability of RAC. Nonetheless, the studies on the damping property of RAC are still very few.

So far, most reported experimental investigations and numerical analyses concerning stress amplitude-dependent damping show that high stress amplitude has a very significant influence on damping. However, few studies have focused on the stress amplitude-dependent damping function of widely used concrete materials at low stress amplitudes, especially for recycled aggregate concrete. As a refined constitutive model is increasingly important for the safety and serviceability assessment of RC structures during vibration induced by wind or earthquake, damping at low stress amplitudes should be discussed in detail. In this study, an expression for the loss factor and bending stress amplitude was established based on Lazan’s damping-stress function, and an experimental investigation was conducted to study the loss tangent of RAC at a low stress amplitude. In addition, the damping energy coefficient and exponent used to determine the stress amplitude-dependent damping function of RAC were obtained by numerical fitting.

## 2. Theoretical Derivation

### 2.1. Material Damping Models

Lazan’s damping-stress function is given as

(1) $$D_{e}=J{\text{σ}}_{a}^{n}$$

where $D_{e}$ is the damping energy per cycle for a unit volume of material; ${\text{σ}}_{a}$ is the stress; J and n are the damping energy coefficient and exponent, respectively.

Theodorsen and Garrick’s complex damping model [36] is convenient for describing viscoelastic material behavior, and its damping parameters can be easily determined experimentally. This damping model is therefore widely used, and it is given as

(2) $$\sigma =E^{*}\varepsilon =(E+iE^{\prime \prime })\varepsilon =(1+i\eta )E\varepsilon$$

where $E^{*}$, E, and $E^{\prime \prime }$are the complex modulus, elastic modulus, and loss modulus, respectively; η is the loss tangent; σ, ε are the bending stress and strain, respectively.

### 2.2. Member Model and Assumptions

**Figure 1:**
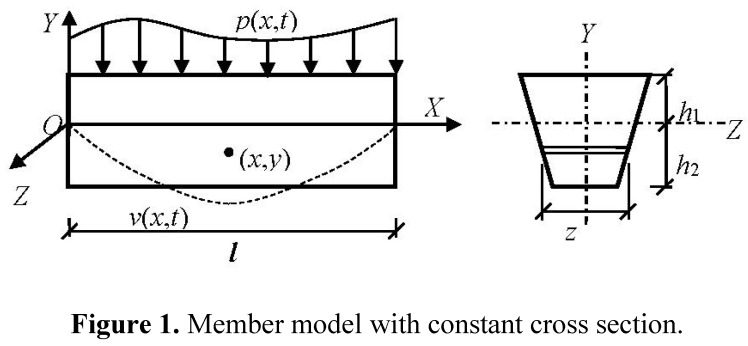
Member model with constant cross section.

Figure 1 shows the member model with a constant cross section, and its boundary conditions are arbitrary. The following assumptions are made:

(1) The member is only loaded in the Y direction and bent in the XOY plane. The load types are arbitrary.
(2) The cross section is symmetric about the Y-axis, and the bending stress is uniform along the Z-axis of the cross section. The cross section shape is arbitrary.
(3) The transverse bending vibration is the member’s main vibration mode, and the influence of shear stress on damping is neglected.
(4) The material is considered continuous and isotropic, and the bending stress amplitude is low.

### 2.3. Elastic Strain Energy of Member

The elastic strain energy for a unit volume of the member $U_{e}$, as shown in Figure 1, is defined as

(3) $$U_{e}=\frac{{{\left(\sigma_{ax}\right)}_{y}}^{2}}{2E}$$

where ${\left(\sigma_{ax}\right)}_{y}$ is the bending stress at coordinates (*x*, *y*); *y*, the distance from the neutral axis of the member’s cross section; and *x*, the coordinate along the longitudinal axis of the member. The elastic strain energy of the global member $U_{s}$ is obtained as

(4) $$U_{s}=\int_{V}U_{e}\text{d}V=\iint_{\Omega }U_{e}z\text{d}x\text{d}y=\left(\frac{\sigma_{am}^{2}}{2E}\right)\left(lA\right)\left(\frac{1}{A}\int_{-h_{2}}^{h_{1}}y^{2}zh_{i}^{-2}\text{d}y\right){\left(\frac{1}{l}\int_{0}^{l}{\left(\frac{\sigma_{ax}}{\sigma_{am}}\right)}^{2}\text{d}x\right)}_{l}$$

where *A* is the member’s cross-sectional area; *l* is the effective length of the member; *h*_1_ and *h*_2_ are the distance from the top and bottom edge to the neutral axis, respectively; *h_i_* is the greater of *h*_1_ and *h*_2_; *z* is the width of the cross section at coordinates (*x*, *y*);

$${\text{σ}}_{ax}={\left({\text{σ}}_{ax}\right)}_{y}\times h_{i}/y$$

is the maximum bending stress amplitude of the cross section at coordinate *x*; and $\sigma_{am}$ is the maximum bending stress of the whole member.

Moreover, we define

(5) $$U_{am}=\frac{{\text{σ}}_{am}^{2}}{2E}\;,\;V_{s}=lA\;,\;\chi_{c}=\frac{1}{A}\int_{-h_{2}}^{h_{1}}y^{2}zh_{i}^{-2}\text{d}y,\text{ and }\chi_{l}=\frac{1}{l}\int_{0}^{l}{\left(\frac{{\text{σ}}_{ax}}{{\text{σ}}_{am}}\right)}^{2}\text{d}x$$

where $U_{am}$ is the maximum elastic strain energy; $V_{s}$ is the entire volume; $\chi_{c}$ and $\chi_{l}$ are the cross section factor and length factor for the member’s elastic strain energy, respectively. Then, the elastic strain energy of the global member can be simply written as

(6) $$U_{s}=U_{am}V_{s}\chi_{c}\chi_{l}$$

### 2.4. Damping Energy of Member

According to Equation (1), the damping energy of a unit volume at coordinates (*x*, *y*) can be expressed as

(7) $$D_{e}=J{\left({\text{σ}}_{ax}\right)}_{y}^{n}$$

The damping energy of the global member *D_s_* is obtained as

(8) $$D_{s}=\int_{V}D_{e}\text{d}V=\iint_{\Omega }D_{e}z\text{d}x\text{d}y=\left(J{\text{σ}}_{am}^{n}\right)\left(Al\right)\left(\frac{1}{A}\int_{-h_{2}}^{h_{1}}y^{n}zh_{i}^{-n}\text{d}y\right)\left(\frac{1}{l}\int_{0}^{l}{\left(\frac{{\text{σ}}_{ax}}{{\text{σ}}_{am}}\right)}^{n}\text{d}x\right)$$

We define

(9) $$D_{am}=J{\text{σ}}_{am}^{n},\;\gamma_{c}=\frac{1}{A}\int_{-h_{2}}^{h_{1}}y^{n}zh_{i}^{-n}\text{d}y,\text{ and }\gamma_{l}=\frac{1}{l}\int_{0}^{l}{\left(\frac{{\text{σ}}_{ax}}{{\text{σ}}_{am}}\right)}^{n}\text{d}x$$

where $D_{am}$ is the maximum damping energy for unit volume of the member, and $\gamma_{c}$ and $\gamma_{l}$ are the cross section factor and length factor for the member’s damping energy, respectively. Then, the damping energy of the global member takes the form

(10) $$D_{s}=D_{am}V_{s}\gamma_{c}\gamma_{l}$$

### 2.5. Loss Factor of Member

The member’s loss factor $\eta_{s}$ is defined as

(11) $$\eta_{s}=\frac{D_{s}}{2\pi U_{s}}$$

By substituting Equations (6) and (10) into Equation (11) and taking into account Equations (5) and (9), the member’s loss factor can be rewritten as

(12) $$\eta_{s}=\frac{JE}{\pi }{\text{σ}}_{am}^{n-2}\psi_{c}\psi_{l}$$

where $\psi_{c}$ and $\psi_{l}$ are the cross section factor and length factor for the member’s loss factor, and $\psi_{c}$ and $\psi_{l}$ are the ratios of $\gamma_{c}/\chi_{c}$ and of $\gamma_{l}/\chi_{l}$, respectively.

The values of $\psi_{c}$ for some special cross sections are listed in Table 1, and the values of $\psi_{l}$ for some boundary conditions with special loads are presented in Table 2. All values of $\psi_{c}$ and $\psi_{l}$ are equal to 1.0 when *n* = 2, which proves the correctness of these expressions of $\psi_{c}$ and $\psi_{l}$.

**Table 1:** Values of $\psi_{c}$ for some special cross sections.

Cross Section Forms	Values of $\psi_{c}$
Rectangular Section	$\frac{3}{n+1}$
Circular Section	$\frac{16}{\pi }\int_{0}^{\frac{\pi }{2}}\sin^{n}\theta \cos^{2}\theta \text{d}\theta$
Symmetrical I-section	$\frac{3}{n+1}[\frac{\frac{b_{f}}{b_{f}-b}-{\left(\frac{h-2h_{f}}{h}\right)}^{n+1}}{\frac{b_{f}}{b_{f}-b}-{\left(\frac{h-2h_{f}}{h}\right)}^{3}}]$
T-section	$\frac{3}{n+1}{\left(\frac{2}{h}\right)}^{n-2}\frac{\left[b_{f}h_{1}^{n+1}+bh_{2}^{n+1}-(b_{f}-b){\left(h_{1}-h_{f}\right)}^{n+1}\right]}{\left[b_{f}h_{1}^{3}+bh_{2}^{3}-(b_{f}-b){\left(h_{1}-h_{f}\right)}^{3}\right]}$

Note: *b_f_* and *b* are the flange width and web width, respectively, of Symmetrical I-section or T-section; and *h_f_* and *h* are the flange height and total height, respectively, of Symmetrical I-section or T-section.

**Table 2:** Values of $\psi_{l}$ for some boundary conditions with special loads.

Member’s Boundary Conditions	Load Types	Values of $\psi_{l}$
Simply Supported	Concentrated Load at Mid-span	$\frac{3}{n+1}$
Simply Supported	Uniformly Distributed Load	$\frac{3.75\times 4^{n}}{l^{2n+1}}\int_{0}^{\frac{l}{2}}{\left(lx-x^{2}\right)}^{n}\text{d}x$
Cantilever	Concentrated Load at Cantilever End	$\frac{3}{n+1}$
Cantilever	Uniformly Distributed Load	$\frac{5}{2n+1}$
One End Clamped and Another End with Sliding Bearing	Concentrated Load at the End of Sliding Bearing	$\frac{3}{n+1}$

When n≠2.0, it is obvious that the loss factor of the member is not only dependent on the stress amplitude but also affected by factors such as cross section forms, load types, loading positions, and boundary conditions. This indicates that the loss factor is nonlinear. Conversely, when n=2.0, the exponent of stress amplitude for the loss factor is equal to zero and the values of $\psi_{c}$ and $\psi_{l}$ are equal to 1.0; therefore, the loss factor is independent of the above factors. In this case, the loss factor is linear and Equation (12) is simplified as follows:

(13) $$\eta_{s}=JE/\pi$$

### 2.6. Loss Tangent of Material

Based on the complex damping model and only considering the material’s internal damping, the bending vibration equation for the member, as shown in Figure 1, is obtained by dynamic analysis as reported in [37]

(14) $$\bar{m}\frac{\partial^{2}v(x,t)}{\partial t^{2}}+\left(EI_{\text{c}}+iE^{\prime \prime }I_{\text{c}}\right)\frac{\partial^{4}v(x,t)}{\partial x^{4}}=p(x,t)$$

where $\bar{m}$ is the member’s line density; $I_{c}$, the moment of inertia for the member’s cross section; t, the time; v(x,t), the member’s bending vibration displacement; and p(x,t), the distributed load along the *Y*-axis direction.

The member’s loss factor can be described differently as

(15) $$\eta_{s}=\frac{D_{s}}{2\pi U_{s}}=\frac{E^{\prime \prime }I_{\text{c}}}{EI_{\text{c}}}=\eta$$

By substituting Equation (12) into Equation (15), the loss tangent of the material can be expressed in the form

(16) $$\eta =\frac{JE}{\pi }{\text{σ}}_{am}^{n-2}\psi_{c}\psi_{l}$$

## 3. Experimental Investigation

### 3.1. Materials

#### 3.1.1. Cement

The cement used in this study was ordinary Portland cement with a 28-day nominal compressive strength of 42.5 MPa. The chemical composition of cement is shown in Table 3.

**Table 3:** Chemical composition (by mass) of cement.

SiO_2_ (%)	Al_2_O_3_ (%)	Fe_2_O_3_ (%)	CaO (%)	R_2_O (%)	Loss on Ignition (%)
18.73	5.88	3.37	61.89	0.54	3.03

#### 3.1.2. Fine Aggregate

The natural fine aggregate (NFA) used was river sand and it was controlled by grading to 5 mm. Its apparent density was 2514 kg/m^3^, and its moisture content was 0.96%.

#### 3.1.3. Coarse Aggregate

The natural coarse aggregate (NCA) was common crushed stone, and the recycled coarse aggregate (RCA) was produced from demolished laboratory concrete samples by crushing artificially. The NCA and RCA were controlled by grading to 20 mm, and their properties are listed in Table 4.

**Table 4:** Properties of coarse aggregates.

Types	Apparent Density (kg/m^3^)	Bulk Density (kg/m^3^)	Moisture Content (%)	Water Absorption (%)	Crushing Index (%)
NCA	2561	1380	0.41	1.06	9.8
RCA	2509	1253	2.19	5.48	15.6

#### 3.1.4. Modified Materials

In order to improve the damping, strength, and ductility of the recycled aggregate concrete, modified materials such as polypropylene fiber (PF), steel fiber (SF), rubber powder (RP), micro-silica fume (MSF), ultrafine slag (US), and fly ash (FA, II-grade) were used. The length of PF was 19 mm and its density was 0.91 g/cm^3^; the elastic modulus and tensile strength of PF exceeded 3700 MPa and 650 MPa, respectively. The length of SF was 30 mm. The ratio of length/diameter was 43 and the PF’s tensile strength exceeded 600 MPa. The specific surface area of US was 910 m^2^/kg. The RP used in this study was recycled from waste tires, and its average size was 0.84 mm and its elastic modulus was about 6 MPa. The FA was produced from a power plant and its fineness was 1.78; the SO_3_ content and CaO content were 0.5% and 1%, respectively. The average size of MSF was 0.21–0.26 μm, the SiO_2_ content of MSF was greater than 96% and its 28-day pozzolanic activity index was 90%.

### 3.2. Specimen Preparation

Six types of specimens were designed, and they were classified into two groups as follows:

(1) The first group had three types of specimens, and it was used to study the effect of the RCA replacement ratio on the damping of concrete. Three RCA replacement ratios were considered, *i.e.*, 0%, 30%, and 70%, and corresponding specimens were denoted as RCA-0, RCA-30, and RCA-70, respectively.
(2) The second group also had three types of specimens, and it was used to study the effect of modified materials on the damping of concrete. The specimens were respectively modified by MSF + PF, RP + SF, and FA + US, and the corresponding specimens were denoted as RCA-MSF + PF, RCA-RP + SF, and RCA-FA + US, respectively.

Owing to the maximum exciting force controlled by the existing electromagnetic exciter being limited, the specimens cannot be molded with the same scale as real building members. Therefore, they were designed as 80 mm (width) × 80 mm (height) × 1000 mm (length) beams. Table 5 presents the composition of RAC. The designed water/cement ratio was 0.5 for all proportion cases, and the additional water and water reducer were used to reach the target slump of 30 mm. The additional water was used for considering the high water absorption ratio of RCA, as shown in Table 4, and the water reducer was used to consider the high ratio of water demand of MSF, RP, and US. For each proportion case, two RC beams and six 100 mm cubic blocks were cast. These beams are prepared for the damping test and the cubic blocks are used to assess the 28-day compressive strength and split tensile strength.

**Table 5:** Mixture proportions of recycled aggregate concretes (kg/m^3^).

Types	Cement	Water	Sand	NCA	RCA	Modifiers	AW	Water Reducer
RCA-0	410	205	625	1160	0	0	0	0
RCA-30	410	205	625	812	348	0	19	0
RCA-70	410	205	625	348	812	0	44	0
RCA-MSF + PF	390	205	625	0	1160	20(MSF) + 1.2(PF)	134	7.37
RCA-RP + SF	410	205	575	0	1040	50(RP) + 120(SF)	62	3.0
RCA-FA + US	287	205	625	0	1160	82(FA) + 41(US)	50	1.0

### 3.3. Testing Method

**Figure 2:**
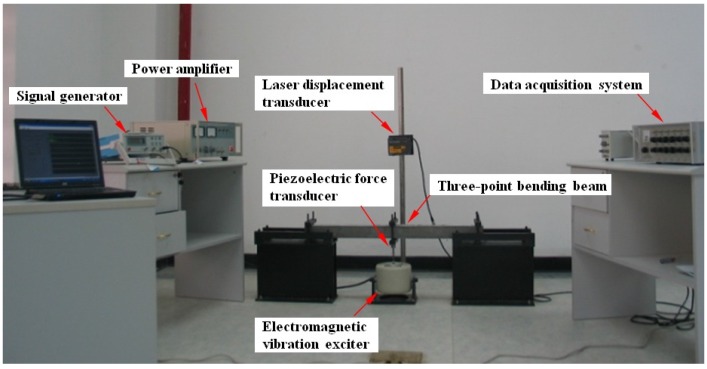
Three-point dynamic bending testing device.

The test was conducted on a three-point dynamic bending testing device, as shown in Figure 2. The testing procedure was led as follows: First, a 1.0 Hz sine signal was produced by a signal-generator. Second, four different exciting force amplitudes (*i.e*., 50, 100, 150, and 200 N) were applied at the middle of the simply supported beam by an electromagnetic vibration exciter. Third, the force value was measured by a force transducer that was fixed under the beam, and the middle point displacement of the beam was measured by a laser transducer above the beam. Finally, all measured data were stored in a computer by a data acquisition system.

Based on the complex damping model, the loss tangent and elastic modulus of RAC can be calculated by Equations (17) and (18) [38].

(17) $$\eta =\frac{1}{1+\frac{v_{0}}{p_{0}\cos\delta }\frac{\omega^{2}\bar{m}l}{2}}\times \tan\delta$$

(18) $$E=\left(\frac{p_{0}}{v_{0}}\cos\delta +2\pi^{2}f^{2}\bar{m}\right)\times \frac{2l^{3}}{I_{\text{c}}\pi^{4}}$$

where $p_{0}$ is the exciting force amplitude; $v_{0}$, the midpoint displacement amplitude of the beam; δ, the phase shift between the exciting force and the midpoint displacement; ω, the first natural radian frequency of the beam; f, the exciting frequency.

### 3.4. Experimental Results and Discussion

#### 3.4.1. Compressive Strength and Split Tensile Strength

The 28-day compressive strength and split tensile strength are presented in Table 6. Compared with the compressive strength of RCA-0, those of RCA-30, RCA-70, RCA-MSF + PF, RCA-RP + SF, and RCA-FA + US were decreased by 3.2%, 6.8%, 39.7%, 50.6%, and 27.4%, respectively. Compared to the split tensile strength of RCA-0, those of RCA-30, RCA-70, RCA-MSF + PF, RCA-RP + SF, and RCA-FA + US were also decreased by 4.1%, 9.1%, 28.6%, 4.1%, and 29.7%, respectively.

The old mortar adhered on the surface of RCA has a large number of pores and micro-cracks produced by crushing, which lead to low density and high absorption. As a result, the crushing index and water absorption of RCA are increased by 59.2% and 417%, respectively, compared with that of NCA, shown in Table 4. Thus, the compressive strength and split tensile strength both decreased with an increase in the RCA replacement ratio. The modified recycled aggregate concretes with more RCAs were supplemented with water reducer, which increased the effective equivalent water/cement ratio, and their compressive strength decreased significantly. Owing to the low elastic modulus of RP, the compressive strength of RCA-RP + SF was lowest, but the split tensile strength of RCA-RP + SF was almost equal to that of RCA-0 due to the reinforcement of SF.

**Table 6:** The 28-day compressive strength and split tensile strength.

Specimens	RCA-0	RCA-30	RCA-70	RCA-MSF + PF	RCA-RP + SF	RCA-FA + US
Compressive Strength/Standard deviation (MPa)	50.4/1.6	48.8/1.8	47.0/0.4	30.4/1.5	24.9/1.1	36.6/1.4
Split Tensile Strength/Standard deviation (MPa)	5.36/0.45	5.14/0.14	4.87/0.38	3.83/0.34	5.15/0.40	3.77/0.27

#### 3.4.2. Loss Tangent and Elastic Modulus

The loss tangent and elastic modulus of RAC are shown in Table 7. It is found that the loss tangent for all types of RAC increases with the exciting force amplitude even if the beams are under low-amplitude vibrations. Compared with the loss tangents at the exciting force amplitude of 50 N, the loss tangents at the exciting force amplitude of 200 N increased by 5.8% (RCA-0), 3.9% (RCA-30), 6.0% (RCA-70), 20.1% (RCA-MSF + PF), 16.0% (RCA-RP + SF), and 13.6% (RCA-FA + US), respectively.

The loss tangent increases slightly with the RCA replacement ratio and increases significantly when the RAC was added with modified admixtures. Compared with the loss tangent of RCA-0 at the exciting force amplitude of 200 N, the loss tangents increased by 1.2% (RCA-30), 5.5% (RCA-70), 20.1% (RCA-MSF + PF), 71.0% (RCA-RP + SF), and 47.4% (RCA-FA + US), respectively.

The elastic modulus at the exciting force amplitude of 200 N is reduced by 5.1% (RCA-70), 16.7% (RCA-MSF + PF), 16.2% (RCA-RP + SF), and 13.6% (RCA-FA + US), compared with that of RCA-0. In general, the elastic modulus decreased slightly with the RCA replacement ratio, and decreased markedly when the RAC was added with modified admixtures. However, no obvious regularity is found about the relationship between the elastic modulus and the exciting force amplitude at low-amplitude vibrations.

**Table 7:** The loss tangent and elastic modulus of recycled aggregate concrete.

Specimens	Loss Tangent (%)				Elastic Modulus (10^4^ N/mm^2^)			
	50 N	100 N	150 N	200 N	50 N	100 N	150 N	200 N
RCA-0	5.50	5.63	5.66	5.82	3.68	3.55	3.36	3.54
RCA-30	5.67	5.71	5.83	5.89	3.63	3.55	3.49	3.54
RCA-70	5.79	5.93	6.00	6.14	3.00	3.30	3.27	3.36
RAC-MSF + PF	5.82	6.10	6.62	6.99	2.92	3.03	3.02	2.95
RAC-RP + SF	8.58	9.31	9.72	9.95	2.77	2.87	2.75	2.97
RAC-FA + US	7.55	7.88	8.28	8.58	3.59	3.08	3.29	3.06

#### 3.4.3. Energy Coefficient and Exponent

When a concentrated force with amplitude $p_{0}$ is applied at the mid-span of a simply supported beam with a rectangular cross section, the maximum elastic stress of the beam is

(19) $${\text{σ}}_{am}=\frac{3p_{0}l}{2b_{\text{r}}{h_{\text{r}}}^{2}}$$

where $b_{r}$ and $h_{r}$ are the width and height of the rectangular cross section, respectively. By substituting Equation (19) and the values of $\psi_{c}$ and $\psi_{l}$ into Equation (16), the loss tangent takes the form

(20) $$\eta =\frac{9JE}{\pi {\left(n+1\right)}^{2}}{\left(\frac{3p_{0}l}{2b_{\text{r}}{h_{\text{r}}}^{2}}\right)}^{n-2}$$

The numerical fitting for the relationship between the loss tangent and the exciting force amplitude was performed according to Equation (20) by Matlab programming, and the damping energy coefficient and exponent of RAC are obtained, as shown in Table 8.

**Table 8:** Damping energy coefficient and exponent of recycled aggregate concrete.

Specimens	Damping Energy Coefficient *J* (×10^−6^ MPa^−1^)	Damping Energy Exponent *n*	Correlation Coefficient *R*^2^
RCA-0	5.39	2.04	0.862
RCA-30	5.41	2.03	0.898
RCA-70	6.29	2.04	0.957
RCA-MSF + PF	9.53	2.10	0.969
RCA-RP + SF	13.04	2.14	0.996
RCA-FA + US	8.73	2.14	0.937

The influence of the RCA replacement ratio on the damping energy exponent is slight, and the value of n is about 2.04. The influence of modified admixtures on the damping energy exponent is obvious, and the value of n is increased to 2.10–2.14. It should be noted that all values of n are not equal to 2.0, indicating that the recycled aggregate concrete is not a type of completely elastic material, as we usually think, even at low bending stress amplitudes.

Compared with the damping energy coefficient of NAC, those of RACs are increased by 0.4% (RCA-30), 16.7% (RCA-70), 76.8% (RCA-MSF + PF), 141.9% (RCA-RP + SF), and 62% (RCA-FA + US), respectively. The damping energy coefficient of the RAC is greater than that of the NAC, implying that the energy dissipation capacity of the RAC is greater than that of the NAC under the same conditions.

## 4. Conclusions

The relationship between the loss factor and the stress amplitude of a member is derived when the member is at a low-amplitude bending vibration. The loss factor of the member depends not only on the stress amplitude, but also factors such as the cross section shapes, boundary conditions, load types, and loading positions.

The loss tangent of RAC is obtained through experimental investigations. The loss tangent increases slightly with the bending stress amplitude and with the RCA replacement ratio, and increases significantly with adding modified admixtures.

The damping energy coefficient and exponent are obtained by numerical fitting to determine an explicit stress amplitude-dependent damping function of RAC. The damping energy exponent of RAC is not equal to 2.0, indicating that the damping of RAC, especially the RAC with modified admixtures, is nonlinear. In addition, compared with NAC, the damping energy coefficient of RAC is increased, and it is further increased by modified admixtures.

## Notation

$\psi_{c}$: the cross section factor for the member’s loss factor
$\psi_{l}$: the length factor for the member’s loss factor
n: damping energy exponent
J: damping energy coefficient, MPa^−1^
E: elastic modulus of material, MPa
$\sigma_{am}$: the maximum stress of the member, MPa

## Appendix

Stress Amplitude-Dependent Damping Function

(A1) $$\eta_{s}=\frac{JE}{\pi }\sigma_{am}^{n-2}\psi_{c}\psi_{l}$$
